# Influence of Recycled High-Performance Aggregate on Deformation and Load-Carrying Capacity of Reinforced Concrete Beams

**DOI:** 10.3390/ma13010186

**Published:** 2020-01-02

**Authors:** Barbara Sadowska-Buraczewska, Danuta Barnat-Hunek, Małgorzata Szafraniec

**Affiliations:** 1 Faculty of Civil Engineering and Environmental Sciences, Bialystok University of Technology, ul. Wiejska 45 A, 15-351 Bialystok, Poland; barbara.sadowska@pb.edu.pl; 2 Faculty of Civil Engineering and Architecture, Lublin University of Technology, ul. Nadbystrzycka 40, 20-618 Lublin, Poland; d.barnat-hunek@pollub.pl

**Keywords:** high-performance recycled aggregates, high performance concrete (HPC), recycled aggregate concrete (RAC), concrete beams, high strength concrete (HSC)

## Abstract

The use of recycled concrete aggregates (RCA) in high performance concrete (HPC) was analyzed. The paper presents the experimental studies of model reinforced concrete beams with a rectangular section using high-performance recycled aggregates. Two variable contents of recycled aggregate concrete were used in this study: 50% and 100%. The experimental analyses conducted as immediate studies concerned the following issues: short time loads-deflection, load-carrying capacity of beams, deformation of concrete, cracks, and long-term loads-deflection. The comparative analysis involves the behavior of beams made of high performance concrete-high strength concrete (HPC-HSC) recycled aggregates with model control elements made of regular concrete based on natural aggregates. The deflection values for the recycled aggregate beams were 20% higher than in the case of the control beams made of HPC-HSC exclusively. Replacement of aggregate with recycled concrete aggregate resulted in a large decrease in the value of these two parameters, i.e., compression strength by about 42% and modulus of elasticity by about 33%.

## 1. Introduction

The modern construction industry should meet the requirements of providing durable and ecological structures. There are a number of factors that prevent concrete structures from meeting the basic standards of sustainable development.

Special attention should be paid to the problems of climate changes, depletion of natural resources, and non-environmentally-friendly cement production. During the cement production process, a large amount of gases is released into the environment, contributing to the global greenhouse effect and climate changes in many parts of the Earth. Moreover, large quantities of particularly valuable natural resources such as gravel, sand, crushed rock aggregate, and water, are used in the course of concrete production. It is estimated that, in order to produce Portland cement, approximately 1,000,000,000 tons of raw materials are used each year.

Over the last 30 years, a rapid development has occurred in terms of designing, optimizing and improving formulas of high-performance concrete. This type of concrete has been increasingly widely used in practice. The future of HPC is high and super-high buildings [1]. Nowadays, many buildings and bridges all over the world are built of high-performance concrete. Owing to the application of the high-performance concrete, more economical and functional solutions are arrived at, and higher durability of concrete reduces the natural resource consumption [2,3,4]. To the best of the authors’ knowledge, few studies focused on the effects of recycled aggregates used in HPC [1,5,6,7,8,9,10].

Another reason for the increased interest in high-performance concrete involves stronger and stronger trends connected to implementation of recycling in construction [11,12]. In addition to the recycling of paper, glass, or metals, well-known so far, there is also the recycling of concrete, leading to obtaining recycled aggregate [13]. The growing ecological awareness of the society results in seeking new solutions in the construction industry [14]. In order for the effects of the environment protection policy to be visible and long-lasting, actions should be taken systematically and the principles of sustainable development should be implemented [15,16].

As a consequence of ecological practices, a growing tendency for the application of recycled materials can be observed in many areas of industry. Using construction and demolition debris as a substitute for natural aggregate in the production of concrete mixtures is gaining popularity due to both economic and ecological reasons. The use of construction and demolition debris means limiting the use of expensive natural aggregate, and thus minimizing the negative impact on the natural environment. The maximum benefit is achieved when the greatest possible amount of recycled aggregate is used in the production of concrete [17,18,19,20,21,22], including HPC [10,23,24,25,26]. Hopefully, the continuous development of technologies in the construction sector will lead to minimizing the amount of waste as well as increasing the amount of materials undergoing the recycling process and reused for building new structures [27,28].

It can be assumed that, because of the growing social emphasis on ecological aspects, the recycled aggregate will be increasingly widely applied as a construction concrete component of full value. Even though HPC is used for building structures, they may have to be demolished at some point in the future. The obtained debris may be reused as recycled aggregate made of high-performance concrete of which the structural elements had been made. The issue pertaining to the reuse of building rubble from demolition or reconstruction has been a long-standing concern in industrialized countries [29,30,31,32]. This paper investigates the possibilities for the maximum application of the debris obtained from demolishing objects made of HPC.

The aspects connected to protecting the natural environment gain importance with time; therefore, the interest in alternative construction solutions has been growing. The idea of reusing concrete debris is increasingly often taken into consideration as people are against establishing another open-pit mine to mine aggregate, since they have a significant negative impact on large areas.

Two fundamental reasons leading to the popularization of recycling are the continuous growth of the amount of concrete waste and the constant increase in the use of concrete as a construction material [14,33].

## 2. Materials and Methods 

### 2.1. Materials

The concrete was prepared using CEM I 52.5 R Portland cement. The technical parameters of applied Portland cement *Górażdże* are presented in Table 1.

**Figure 1:**
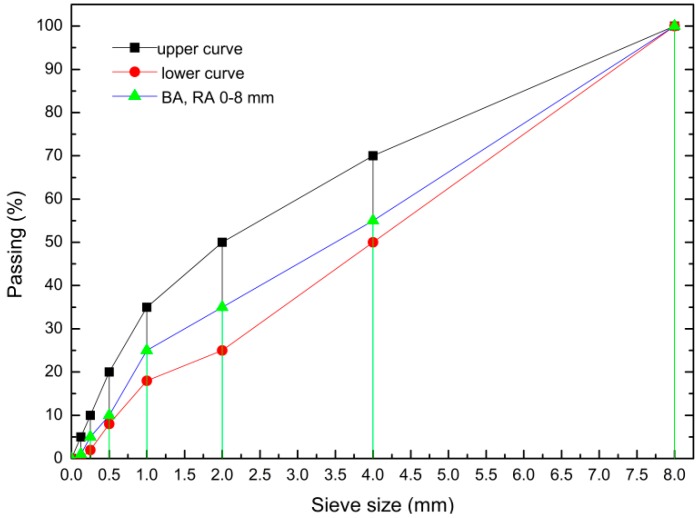
Gradation of basalt and recycled aggregates.

Natural rinsed quartz sand (0–2 mm), natural basalt aggregate (BA) (2–8 mm), recycled aggregate (2–8 mm), and silica fume (SF) were utilized for concrete preparation. The grain size of BA and RA is shown in Figure 1.

Three concrete mixtures were prepared with the composition presented in Table 2. As shown in Table 2, the referenced concrete (HPC-HSC) was made of natural aggregate and the other two concrete compositions varied in the content of recycled aggregate (REC-1 50%, REC-2 100%). A retarding superplasticizer based on polycarboxylic ether has also been added to the concrete.

The chemical composition of the natural quartz is shown in Table 3.

The density of quartz sand amounted to 2.65g∙cm^−3^. The properties of basalt and recycled aggregate are shown in Table 4.

The chemical composition of the basalt is shown in Table 5.

The chemical composition of the silica fume is shown in Table 6.

The density of silica fume amounted to 2.40 g∙cm^−3^. The properties of the superplasticizer are given in Table 7.

### 2.2. Methods

In order to determine the physical properties of each concrete type, 18 cubic samples with an edge length of 150 mm were prepared (6 for each concrete). They were used to determine the apparent density, absorption of water and bulk density. The bulk density was determined according to the PN-EN 12390-7 standard [37]. In order to determine the mechanical properties of concrete, 36 cubic samples with the edge length of 150 mm were prepared for determine the compressive strength: 9 samples were examined after 7 days of ripening of concrete (3 for each concrete), 9 after 14 days of period (3 for each concrete), and the other 18 after 28 days of period (6 for each concrete). In order to determine elastic modulus, cylindrical specimens with a diameter of 150 mm and height of 300 mm were used (6 specimens for each concrete). The bending tensile strength was tested using 9 cuboid samples with the dimensions of 100 mm × 100 mm × 400 mm (3 specimens for each concrete). Carrying capacity, deformations, and deflections for each concrete were tested with 9 cuboid reinforced concrete beams with the dimensions of 80 mm × 120 mm × 1100 mm (3 specimens for each concrete). A long-term deflection test was conducted on 9 cuboid specimens with the dimensions of 40 mm × 40 mm × 500 mm (3 specimens per concrete). 

Concrete porosity was tested by capillary mercury injection (MICP) using AutoPore IV 9520 t (Norcross, GA, USA) to test pores between 2 nm and 200 μm in diameter [38]. Cubic specimens with edge length of 10 mm were randomly cut from a rectangular specimen. The weight of the small pieces of concrete was approximately 2.5 g. Prior to the measurement, all specimens were heated overnight at 105 °C and then degassed under vacuum, which is the standard procedure in the mercury intrusion porosimetry (MIP) analysis [39]. Six samples were prepared for each type of concrete. The surface area of the sample was dry because the presence of other liquids prevents the injection of mercury. The average pore diameter of each concrete was determined as well.

In accordance with the PN-EN 12390-3 standard [40], the compressive strength test was performed on the cubic samples with the edge length of 150 mm. The elastic modulus values were determined based on the 12390-13 standard [41] on the basis of the following Equation (1):

(1) $$E=\frac{\sigma_{G}\;-\;\sigma_{D}}{\left(\frac{\varepsilon^{\prime }{}_{G}\;+\;\varepsilon^{\prime \prime }{}_{G}\;+\;\varepsilon^{\prime \prime \prime }{}_{G}}{3})-(\frac{\varepsilon^{\prime }{}_{D}\;+\;\varepsilon^{\prime \prime }{}_{D}\;+\;\varepsilon^{\prime \prime \prime }{}_{D}}{3}\right)}(\mathrm{GPa}),$$

where ε_G_—deformation at the upper range of the force level, amounting to 250 kN (this value is determined by the maximum pressure force exerted by the universal testing machine used in the research); the measurement base—100 mm, ε_D_—deformation at the lower range of the force level, amounting to 20 kN (this value is determined by the minimum pressure force at which the first noticeable deformations appear); the measurement base—100 mm, σ_G_—stress at the upper range of the force level, σ_D_—stress at the lower range of the force level.

The bending tensile strength test was carried out in accordance with the PN-EN 12390-5 standard [42] on the cuboid samples also conforming to this standard. The samples tested in this experiment (after 28 days of ripening) were placed in the testing machine, and the three-point flex test was conducted. The bending tensile strength was calculated afterwards based on the maximum load.

**Figure 2:**
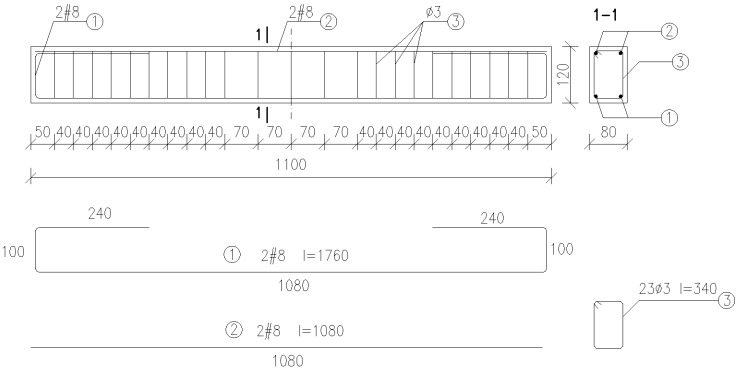
The scheme of reinforcement for beams.

Carrying capacity, deformations and deflections were determined on 9 cuboid reinforced concrete beams with the dimensions of 80 mm × 120 mm × 1100 mm after 28 days of samples maturing (Figure 2). The reinforcement used for the respective batches can be seen in Figure 2. The main reinforcement was made of B500 steel in the form of 4 bars with diameter of #8. The stirrups, made of smooth finish steel, had the diameter of ф 3.

The research elements were placed on the test stand. The tests on the beams were performed using a universal testing machine, with a simple-supported beam as the static diagram. The momentary load consisted of two concentrated forces applied at one-third of the beam spans. After the beams had been placed on the test stand and the appropriate sensors had been set to measure the particular parameters, the measurements began. The force was applied slowly, and it was increased by 5 kN; then, the necessary measurements were conducted. The strength tests of the investigated reinforced concrete beams were performed until their destruction. The deformations of concrete were measured with an extensometer. The measurements were carried out at the two levels: 1 (in the compression zone) and 2 (in the tension zone), each time after the load force value had increased.

The measurements of the deflection of the reinforced concrete beams were carried out using dial gauges with 0.001 mm resolution. The gauges were placed at three locations: on the left support, in the beam midspan, and on the right support. The deflection was measured each time after the force level had been increased by 5 kN. The long-term measurements consisted of measuring the deflection of the bent concrete elements. The aim of this research was to analyze the deformability of the beams under study in the function of time, with the constant loading value. The bars with the dimensions of 40 mm × 40 mm × 500 mm were the test samples for the long-term experiments (3 for each concrete). The test elements were placed in the test stand designed specifically for the purpose of this research. A load was applied to the beams in a room maintained under constant temperature conditions. The deflections of the bars under research were read every day over approximately 100 days.

## 3. Results and Discussion

The physical properties of the recycled aggregate concrete and the HSC-HPC concrete adopted for the examination are shown in Table 8, while the mechanical properties are presented in Table 9 and Table 10.

The use of recycled aggregate resulted in a reduction in the apparent density of REC-1 and REC-2 concrete by 4% and 7%, respectively, compared to the reference concrete. Moreover, a clear influence of the recycled aggregate addition on water absorptivity can be observed; an increase of 18% was recorded for the REC-1 concrete and 49% for REC-2. The use of recycled aggregate has also contributed to an increase in open porosity by 15% for REC-1 and by 38% for REC-2, which affects lower compression strength, as shown in Table 9. In several studies [8,43,44,45], it has been reported that recycled concrete aggregates have a lower density and higher water-absorption capacity. The studies by Pedro et al. show that the change in water absorption by capillarity occurs more intensively in early times and that it increases as natural aggregates are replaced by recycled aggregates [46]. The reference concrete reached a water capillary absorption value of 1.23–2.41 × 10^−3^ g∙mm^−2^ after 72 h, while the recycled concrete with 50% and 100% substitutes, showed a value of 1.68–2.83 × 10^−3^ g∙mm^−2^ and 1.84–3.43 × 10^−3^ g∙mm^−2^ [46], respectively. The water absorption by immersion increased to 80% due to the total replacement of natural aggregates with recycled aggregates.

In the water absorption and sorption tests carried out by Zega and Di Maio, these parameters for recycled aggregate concretes (RACs) were 15% higher than those for the reference concrete, while, in the water penetration test, all concrete types show a similar behavior. This is due to the different water transport mechanisms in each of these concretes [47].

According to Zhang et al. [24], the RAC densities were lower due to much higher porosity of the existing old cement matrix. Therefore, the introduction of recycled aggregate from old concrete directly weakened the bulk density of the prepared new UHPC and thus led to a lower RAC compression strength. In addition, the introduction of aggregates from old concrete may also lead to an increase in the content of the old cement matrix and a corresponding decrease in the content of the new cement matrix by surface or volume, rather than by mass, as the cement dose was the same for all ultra-high performance concrete (UHPC) types. As a result, the overall mechanical properties of the cement matrix in RAC may be weakened, leading to a loss of the compressive strength [24].

**Figure 3:**
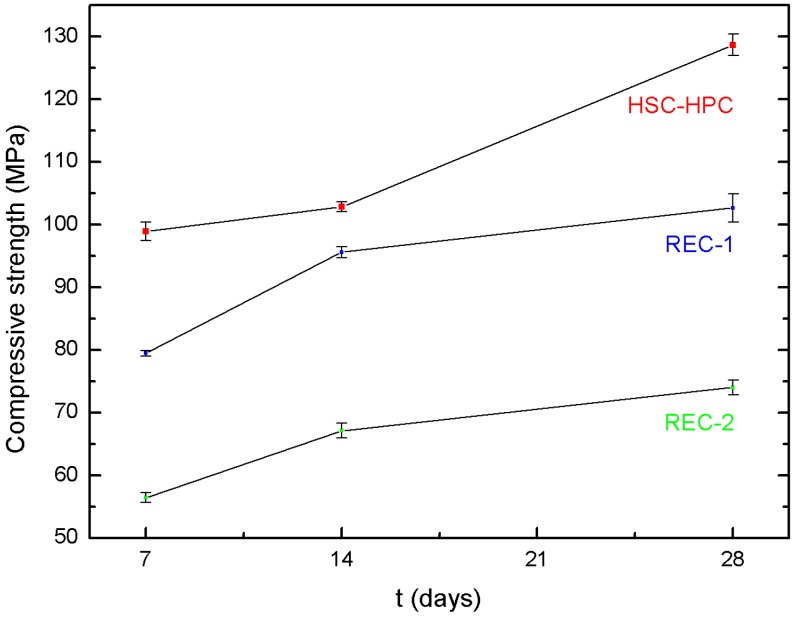
Strength of concretes after 7, 14, and 28 days.

Figure 3 presents the compressive strength values of cubic samples with the edge length of 150 mm after 7, 14 and 28 days of ripening. Figure 3 also indicates that the recycled aggregate content in concrete has an impact on the compressive strength value. The 7-day compression strength studies show that the lowest f_c_ value of 56 MPa is found in the concrete with the highest recycled aggregate content (REC-2 100%). It is 30% lower than for the concrete with 50% recycled aggregate content and as much as 43% lower than for the basalt concrete. After 14 days and after 28 days of ripening of all three concrete types, the basalt concrete still showed the best compression strength results. The worst compression strength after 7, 14, and 28 days was recorded for the concrete with 100% recycled aggregate content, which was 43%, 35%, and 43% lower than the reference concrete, respectively.

Other researchers, e.g., Çakır [49], also noted that, as the amount of recycled aggregate in concrete increases, the strength of the concrete decreases. The research results on the physical and mechanical properties of natural (NAC) and recycled aggregate concrete (RAC) can be observed in the work by Gonzalez-Corominas et al. [8]. They observed that the increase in the aggregate replacement rate had a negative effect on the physical properties of the concrete with recycled aggregate. The modulus of elasticity was also reduced linearly by increasing the amount of recycled aggregate, as well as the physical properties. However, while evaluating the compressive strength, it was observed that the results between the reference concrete and recycled aggregate concrete in 60% and 40% were similar. 

The compression strength of RACs was least dependent on the quality and quantity of RAC than in studies carried out by other authors. After 90 days of concrete ripening, RACs showed higher increases in the compression strength than NAC. According to some authors [17,19], improved phase-to-phase transition zone (ITZ) and internal curing [50] may be responsible for increasing the mechanical strength of RAC. However, it should be noted that Gonzalez-Corominas et al. [8] tested the reference concrete with dolomite and river gravel; hence, they obtained different dependencies than in our article because, in our research, basalt was used as the natural aggregate, which has much higher strength parameters than dolomite and gravel. Therefore, after 28 days, the obtained REC-1 strength was 21%, while REC-2 was 42.6% lower than for HSC-HPC (Table 9, Figure 3). This is consistent with the research of other authors, who also found a reduction in the compression strength by 20–30% of HPC with recycling aggregate in comparison with the HPC with natural aggregate [5,51,52,53,54].

As noted by Zhang et al. [24], f_c_ decreased faster with the increase of the recycling rates of aggregate under autoclaving conditions than under standard conditions of its ripening. In the latest research of Wang et al. [55], the compression strength of the obtained concrete decreased by 8.7–14.0% when the coarse aggregate was completely replaced by recycled aggregate. This decrease in strength is within the ranges from 8.3–23% reported by the majority of authors for RAC with the coefficient of 100% replacement of coarse aggregate by waste aggregate [5,56,57,58].

In the study by Fonseca et al. [9], it was expected that the compression strength would decrease linearly with the replacement of natural aggregate by recycling, as demonstrated by other authors [17]. In fact, the difference between the compressive strength of all analyzed concrete mixes is equal to or less than 7.5%, in relation to NAC. There are various possible reasons; one is that the cement paste attached to the RCA contains non-hydrated cement, which contributes to the increase in strength [27]. Another reason is the increased roughness and specific surface area of RCA, which contributes to a better connection between recycled aggregates and cement paste than the reference concrete [19]. Therefore, the authors could not establish a clear link between the compressive strength and the proportion of recycling opaque in the concrete mix.

As shown by Fonseca et al. [9], the compression strength of all NAC and RAC analyzed by them increased with age. The average compression strength (f_cm_) after 7, 28, and 56 days was 42.8, 49.8, and 51.6 MPa, respectively. In general, after 7 and 28 days of curing, the samples showed about 80% and 95% of their 56-day compression strength [9], respectively. In our study, it was found that, for all concrete types, including HSC-HPC and REC-1 and REC-2, the 28-day strength was 23, 22, and 24% higher than the 7-day strength (see Table 9, Figure 3).

**Figure 4:**
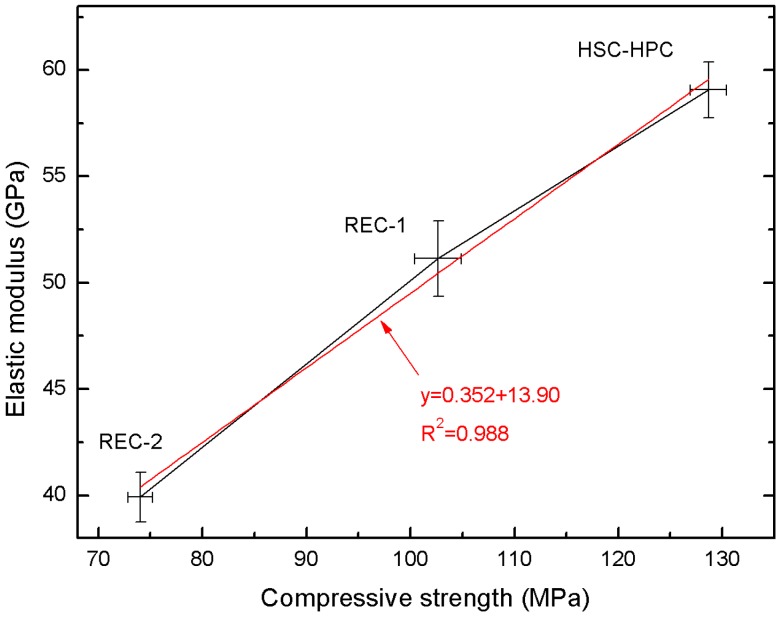
Correlation between compression strength and elastic modulus E of concrete.

Figure 4 presents the changes in the elastic modulus E of concrete depending on the amount of recycled aggregate and concrete compression strength after 28 days. The linear trend was characterized by a R^2^ = 0.988 and relatively low errors in the intercept. The obtained correlations can be described by the equation: y = 0.325x + 13.90.

Flexible modulus of elasticity is closely related to the compressive strength of concrete. The highest parameters were achieved with the concrete comprising the HSC-HPC natural aggregate. Replacement of the aggregate with recycled concrete aggregate resulted in a large decrease in the value of these two parameters—compression strength by about 42% and modulus of elasticity by about 33%.

Concrete is generally considered to be a composite material consisting of three phases: aggregate, cement paste and ITZ [55]. Elastic modulus E of concrete depends mainly on the volume fraction and modulus of elasticity of these three phases [59]. Recycled aggregate is also a kind of composite that consists of natural aggregate, old cement paste, and old ITZ. A numerical model for the elastic modulus of concrete considers the interfacial transition zone [60,61,62]. Therefore, the resulting RAC consists of natural aggregate, old and new cement paste, and old and new ITZ. As shown by Wang [55], the difference between the modulus of elasticity of new cement paste and old cement paste should not be taken into account, since the E_c_ of concrete barely grows after 28 days. E_c_ after 50 years is only 6% higher than E_c_ after 28 days, according to EC2 [63]. The literature analysis by Wang [55] showed that, e.g., in the case of the concrete with an f_c_ value of 45 MPa with a recycled fine aggregate, the modulus of elasticity E varies between 17 GPa and 35 GPa with a 109% difference, while E varies between 15.6 GPa and 36.2 GPa with a 132% difference for coarse recycled aggregate concrete. In addition, the E value decreases by an average of 23% and 32% when the natural aggregate is replaced by the recycled aggregate in 50 and 100%, respectively [55,57,64,65,66].

As shown by Fonseca et al. [9], the 28-day splitting tensile strength of RAC decreased with the increased aggregate content, and it ranged from 2.4 to 3.9 MPa for different RAC incorporation percentages and curing methods. All analyzed RAC concrete had lower values for splitting tensile strength. Different results were obtained by Ajdukiewicz and Kliszczewicz [5], which showed that the tensile strength for mixtures with new waste aggregates is always higher, but the differences are not greater than 10% for 28-day concrete. They proved that the effect of the admixture on the tensile strength is much greater than that of the introduction of recycled aggregate.

Zhang et al. [24], similarly as in the compression strength studies, proved that the flexural strength decreased with the increase in the amount of recycle aggregate, and decreased faster under the autoclave curing.

Ajmani et al. [67] tested the concretes with different contents of recycled aggregate: 20%, 50%, and 80%. The results showed that the splitting tensile strength for R20, R50, and R80 concretes were lower than that of conventional high strength concrete (CHSC) by 74, 90 and 73%, respectively. Lee and Choi [68] also concluded that the value of tensile strength depends on the amount of RA used—the strength decreases as the amount of RA increases.

**Figure 5:**
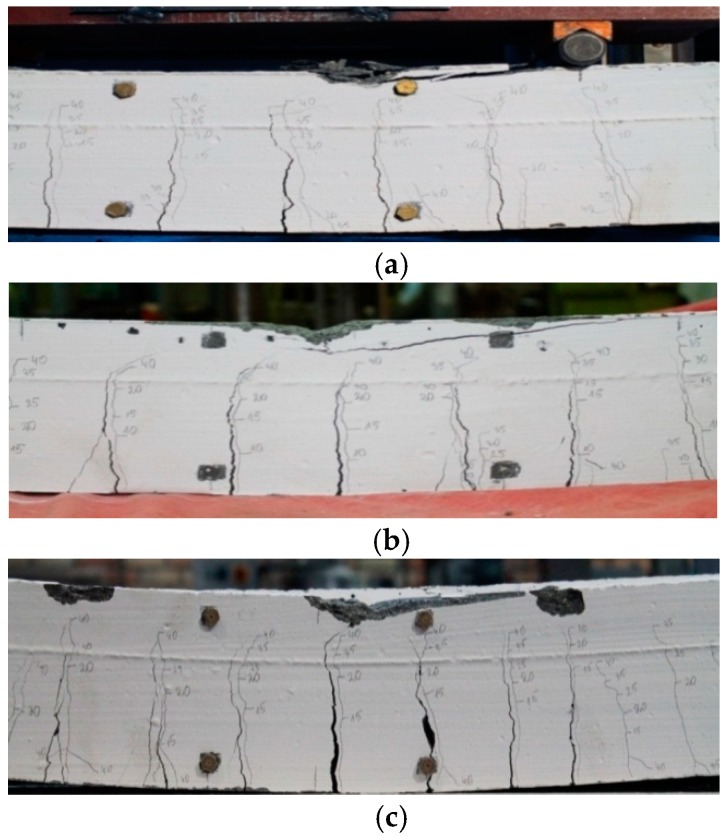
Crack shaping and development in the test samples: (**a**) HPC-HSC; (**b**) REC-1; (**c**) REC-2.

The strength tests of the reinforced concrete beams under investigation were performed until the tested beams were destroyed. At that moment, in each case, a sudden decrease in the rigidity of the beams was observed, as well as a rapid increase in the number of cracks that became much wider, which was indicative of the reinforcement in the tension zone becoming more susceptible to fatigue, and the concrete in the compressed zone was destroyed. The beams were destroyed suddenly, which was accompanied by a characteristic cracking sound. There are many factors that influence the fact that the cracking of reinforced concrete structures is a complex phenomenon. Monitoring of cracking in the reinforced concrete elements is very important because the presence of cracks may indicate the loss of the load-bearing capacity of a structure, which would lead to the concrete being destroyed. Performing the measurements of the width and character of cracks influences the evaluation of the construction safety. The cracking was monitored simultaneously with the deflection and deformation measurements of the tested elements, carried out by the authors of the present paper. Each time, after the loading force had been increased by 5 kN, the appearance and penetration of the formed cracks were marked, with the loading force value inscribed at the end of each crack. The first cracks were observed when the value of the applied loading force was approximately 30% of the critical load, which amounted to 10 kN and 15 kN for the recycled aggregate beams and natural aggregate beams, respectively. Figure 5 presents the development of the formed cracks and the way they were shaped as the loading force for a recycled aggregate beam as well as a natural aggregate beam increased. The concrete beams with basalt showed higher resistance to deformability.

The mean experimental values of rupture force and breaking moments of the recycled aggregate concrete and the HSC-HPC concrete adopted for the examination are shown in Table 11.

**Figure 6:**
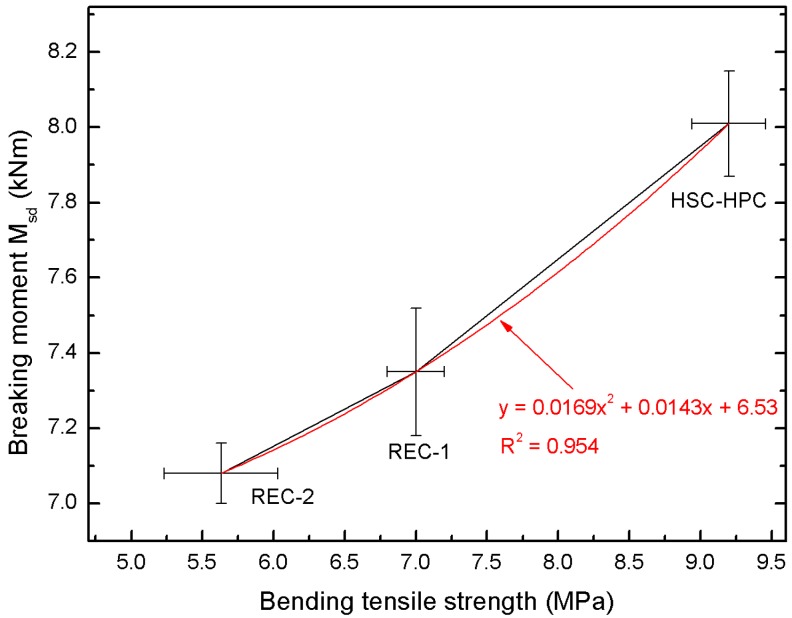
Correlation between bending tensile strength and breaking moment M_sd_ of analyzed concrete.

Figure 6 presents a very good correlation of concrete bending tensile strength in the breaking moment M_sd_.

The correlations described by the polynomial formula y = 0.0169x^2^ + 0.0143x + 6.53 indicate a good coefficient of determination R^2^ = 0.954. Bending tensile strength and breaking moment M_sd_ of concrete also depend on the type and quantity of aggregate used. The best strength parameters were achieved by the concrete with basalt aggregate. As the amount of recycled aggregate in the concrete increases, the bending strength and the breaking moment causing the beams to break down decrease. This moment is approximately 10% lower for the REC-2 concrete than for the HSC-HPC concrete.

**Figure 7:**
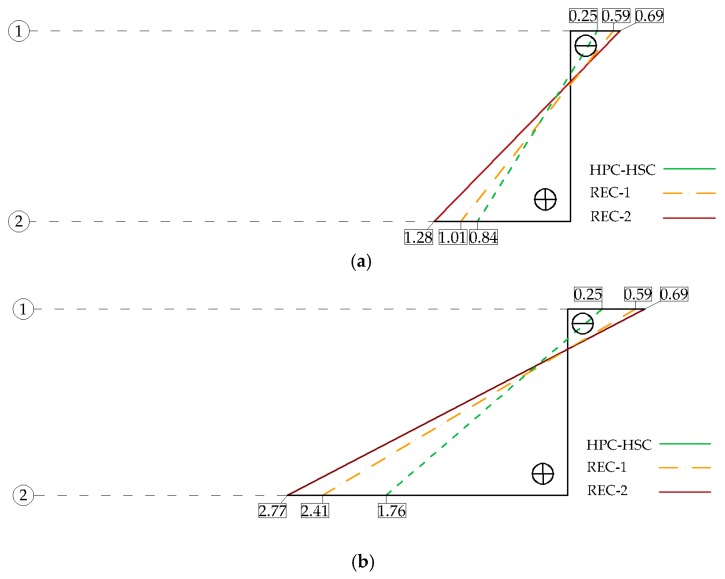

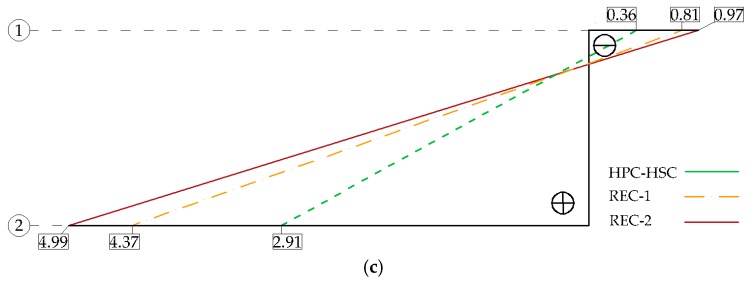
Experimental values of ε (‰) concrete deformations along cross-section height for selected forces: (**a**) force 15kN; (**b**) force 25 kN; (**c**) force 35 kN.

Figure 7 presents the deformations of concrete along the cross-section heights for a few selected force levels.

Having analyzed the above-mentioned results and deformation graphs, it can be concluded that the recycled aggregate beams under study showed greater deformation than the tested natural aggregate beams for the respective values of the loading force. After comparison of the obtained results, it was concluded that the observed mean deformations in the compressed zones of the recycled aggregate beams were, on average, 39% greater than in the case of the natural aggregate beams, while the deformation values in the tension zones were greater by 20% for the recycled aggregate beams in comparison to these for the natural aggregate beams.

**Figure 8:**
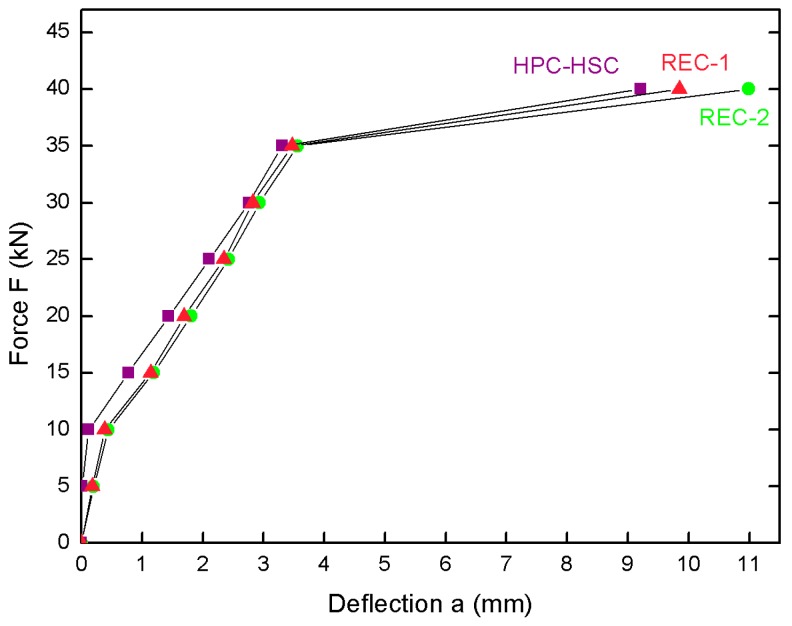
Correlation between force acting on the concrete samples and deflection.

On the basis of the test results shown in Figure 8, it can be stated that the deflection values for the recycled aggregate beams were higher than these for the natural aggregate beams in the case of homogeneous HPC-HSC beams for the given level of the loading force; however, the deflection values were lower in comparison to those for the homogeneous beams made of normal concrete. On average, the deflection values for the recycled aggregate beams were 20% higher than in the case of the control beams made of HPC-HSC alone. It was also noticed that the differences between the deflections observed for the recycled aggregate beams and the natural aggregate beams were significantly greater for the lower loading levels. When the deflection is less than 3 mm, one-way ANOVA analysis of variance at a significance level of 0.05 showed that all means of the deflections results differ statistically significantly between HSC-HPC and REC-1 and HSC-HPC and REC-2, because *p* takes values < 0.05 (0.023, 0.038 respectively). The ANOVA test showed no significant differences in the deflection values between REC-1 and REC-2 (*p* = 0.765).

**Figure 9:**
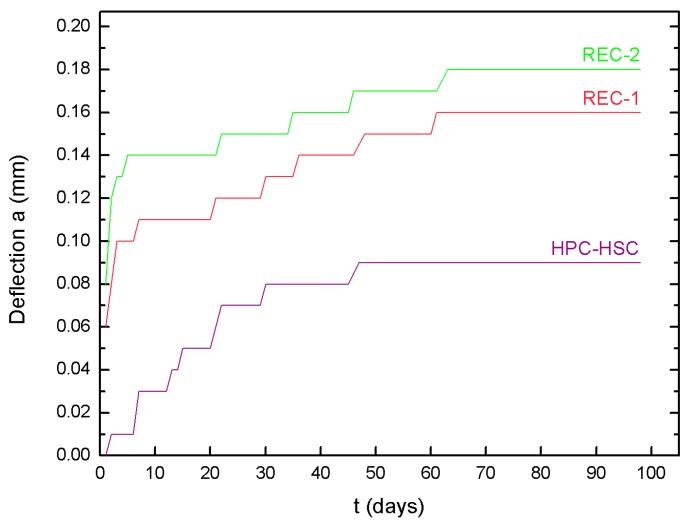
Long-time deflection values for concrete.

Figure 9 presents the dependence of the deflection in the function of time for the recycled aggregate concrete beams and also, for comparative reasons, for the tested homogeneous HPC-HSC beams.

Having analyzed the obtained results of the long-term tests consisting in measuring the deflection of the bent concrete elements, it was concluded that the deflection values of the elements made of the recycled aggregate concrete were, on average, 45% higher than the values of the elements which were made solely of high-performance concrete.

Similar observations on deflections and long-term deflections were made by Łapko A. and Grygo [69]. In their research, they prepared four series of natural aggregate and recycled aggregate beams, which differed in the reinforcement percentage. The studies carried out have shown that the beams with recycled concrete I-R and 1.0% of reinforcement had 42% greater deflections than the beams made of natural aggregates I-N, while the beams II-R with 1.5% of reinforcement showed 57% higher deflections than the beams II-N [69]. Łapko and Grygo also conducted long-term deflections test on the beams of natural dimensions. The results showed that, after 15 weeks, the deflection on the beam made of recycled aggregates was 20% greater than in the beam made of natural aggregates [69]. Ignjatović et al. [70] conducted tests on the beams made of RAC with different aggregate content of recycled aggregate from 50 to 100%, and on the beams made of NAC. Their test showed that the cracking moment of the RAC beams is lower than the cracking moment of the NAC beams, for 10% on average and the RAC beams have 13% higher service deflection. Ajdukiewicz and Kliszczewicz [71] also examined various types of beams (16 series of beams—three elements in each series). Unlike others, they also examined the columns made of recycled aggregate (five series of three columns each series). The studies in [71] showed that the deflections of the RAC beams were about 18% to 100% (at a probable service load) greater than the beams with natural aggregate; the RAC beams have also shown greater deformability. The studies in [72] showed that the deflections of the RC beams with RAC were larger than those with virgin concrete (VC), considering the same moment and same water-to-cement ratio. Sato et al. showed that the RC beams with coarse and fine recycled aggregates (CFRC) exposed to long-term tests, which lasted a year, showed a double increase in deflections compared to the RC beams with VC.

In contrast to others, Łapko and Grygob [73] based their research on the concept of using prefabricated HSC-HPC reinforcement inserts and composed them with recycled aggregate concrete in the most stressed compression zone. The HSC-HPC inserts used in the RC beams made of RAC significantly affect the mechanical properties of beams by increasing flexural stiffness influencing strains and deflections. The reinforced beams tested in [73] showed lower deformations in the range of 20 to 40% compared to the beams made entirely of recycled aggregate.

In several studies [74,75,76,77,78], it was also reported that recycled concrete aggregates have an influence on beam deflections; the recycled aggregate beams were more deformed than the natural aggregate beams.

## 4. Conclusions

The following key conclusions can be drawn from this study:

- Recycled aggregate is viable for use as a substitute of coarse aggregate in concrete structures. Attention has to be paid, however, to the deterioration of some concrete parameters, such as the increased deformability.
- When designing compositions of concrete mixtures with the use of recycled material, a larger safety margin has to be provided for.
- The use of recycled aggregate resulted in a reduction in the apparent density of REC-1 and REC-2 concrete by 4% and 7%, respectively, compared to the reference concrete.
- A clear influence of the recycled aggregate on water absorptivity can be observed; an increase of 18% was recorded for the REC-1 concrete and 49% for REC-2.
- The use of recycled aggregate contributed to an increase in open porosity by 15% for REC-1 and by 38% for REC-2, which affects the lower compression strength.
- Replacement of aggregate with the recycled concrete aggregate resulted in a large decrease in the value of these two parameters—in the compression strength by about 42% and modulus of elasticity by about 33%.
- As the amount of recycled aggregate in the concrete increases, the bending strength decreases.
- The deflection values for the recycled aggregate beams were 20% higher than in the case of the control beams made solely of HPC-HSC.
- The long-term tests consisting of measuring the deflection of the bent concrete elements, showed that the deflection values of the elements made of recycled aggregate concrete were on average 45% higher than the values of the elements, which were made of high-performance concrete exclusively.
- Replacement of basalt with recycled aggregate resulted in the changes in the concrete structure, which affects its physical and strength parameters.
- It can be assumed that the recycled aggregate will be increasingly commonly used as a construction concrete component of full value because of the increasing social emphasis on the ecological aspects.
- Hopefully, the continuous development of technologies in the construction sector will lead to a reduction in the amount of waste produced as well as an increase in the number of materials which can be recycled and reused in concrete manufacturing.

## Figures and Tables

**Table 1:** Technical parameters of CEM I 52.5R Portland cement [34].

Parameters	Unit	Value
Specific surface	(cm^2^∙g^−1^)	4530
Initial setting time	(min)	157
Loss on ignition by weight cement	(%)	3.10
Compressive strength		
after 2 days	(MPa)	35.8
after 28 days		63.7
Density	(g∙cm^−3^)	3.14
Volume stability	(mm)	0.20
SO_3_ content	(%)	2.87
Cl content	(%)	0.049
Insoluble residue	(%)	0.64
Na_2_O_eq_ content	(%)	0.65

**Table 2:** Composition of concrete mixtures.

Components	Unit	HPC-HSC	REC-1	REC-2
CEM I 52.5R cement	(kg∙m^−3^)	450	450	450
Quartz sand (0–2 mm)	(kg∙m^−3^)	630	630	630
Basalt (2–8 mm)	(kg∙m^−3^)	1070	535	–
Recycled aggregate(2–8 mm)	(kg∙m^−3^)	–	535	1070
Silica fume	(kg∙m^−3^)	45	45	45
Superplasticizier	(kg∙m^−3^)	8.1	8.1	8.1
Water	(kg∙m^−3^)	130.9	130.9	130.9

**Table 3:** Chemical composition of the natural quartz sand.

Compositions	SiO_2_	Al_2_O_3_	Fe_2_O_3_	CaO
**Unit** (vol.%)	95.2	2.0	0.6	0.45

**Table 4:** Properties of basalt and recycled aggregates (own research).

Properties	Basalt	Recycled Aggregate
Specific density (g∙cm^−3^)	2.93	2.80
Bulk density (g∙cm^−3^)	2.92	2.60
Absorptivity (%)	0.31	0.78
Abrasion resistance (mm^3^)	6520	4520
Tightness (%)	0.996	0.928
Thermal resistance (°)	340	290
Compressive strength (MPa)	280	155
Frost resistance (F)	F_2_	F_2_

**Table 5:** Chemical composition of the basalt and recycled aggregate (%) (own research).

Compositions	SiO_2_	Al_2_O_3_	FeO	MgO	CaO	TiO_2_	K_2_O	Other Alkaline Compounds
**RA**	26.60	23.22	2.40	2.49	35.25	0.33	3.21	6.5
**BA**	48.5	13.8	10.5	12.2	10	0.9	0.1	4.0

**Table 6:** Chemical composition of the silica fume [35].

Compositions	SiO_2_	Al_2_O_3_	Fe_2_O_3_	CaO	SO_3_	Na_2_O	K_2_O	Other Alkaline Compounds
**Unit** (vol.%)	90	0.4	0.4	1.6	0.4	0.5	2.2	1.9

**Table 7:** Properties of the superplasticizer [36].

Properties	Superplasticizer
Aspect	Light Brown liquid
Relative density at 25 °C (g∙cm^−3^)	1.08
pH	≥ 6
Chloride ion content (%)	<0.2
Expected water reduction (%)	> 20

**Table 8:** Properties of recycled aggregate concrete and the HSC-HPC concrete.

Type of Concrete	Apparent Density (kg∙m^−3^)	Absorptivity (%)	Open Porosity (%)	Average Pore Diameter (µm)
HSC-HPC	2560	1.26	5.54	0.041
REC-1	2450	1.54	6.53	0.055
REC-2	2390	2.48	8.98	0.060

**Table 9:** Characteristics of the recycled aggregate concrete and the HSC-HPC concrete.

Type of Concrete/Descriptive Statistics		Compressive Strength (MPa) f_c,cube#150_			Tensile Strength in Bending (MPa)	Elastic Modulus (GPa)
		after 7 days	after 14 days	after 28 days		
**HSC-HPC**	Mean	98.92	102.85	128.65	9.20	59.07
	SD	1.50	0.81	1.72	0.26	1.31
	CV	1.51	0.79	1.34	2.85	2.22
**REC-1**	Mean	79.47	95.6	102.67	7.00	51.13
	SD	0.46	0.89	2.25	0.20	1.77
	CV	0.58	0.93	2.19	2.86	3.47
**REC-2**	Mean	56.43	67.12	74.03	5.63	39.92
	SD	0.78	1.18	1.20	0.40	1.18
	CV	1.39	1.76	1.62	7.17	2.95

Coefficient of variation—CV (%), Standard deviation—SD.

**Table 10:** Characteristics of the recycled aggregate concrete and the HSC-HPC concrete.

Strength Properties	HSC-HPC	REC-1	REC-2
Class according to PN-EN 206 standard [48]	C100/115	C90/105	C55/67

**Table 11:** Mean experimental values of rupture force and breaking moments.

Symbol of Beam/Descriptive Statistics		Rupture Force F, kN	Breaking Moment M_sd_, kNm
HSC-HPC	Mean	48.00	8.01
	SD	1.73	0.14
	CV	3.61	1.69
REC-1	Mean	43.03	7.35
	SD	1.17	0.17
	CV	2.71	2.38
REC-2	Mean	42	7.08
	SD	1.49	0.08
	CV	3.55	1.08

Coefficient of variation—CV (%), Standard deviation—SD.
